# Transfusion Related Acute Lung Injury (TRALI) Caused by Red Blood Cell Transfusion Involving Residual Plasma Anti-HLA Antibodies: A report on two Cases and General Considerations

**DOI:** 10.1080/17402520500307926

**Published:** 2005-12

**Authors:** Hélène Odent-Malaure, Fabienne Quainon, Pascale Ruyer-Dumontier, Soizick Ducroz, Philippe Verdier, Evelyne Voitellier, Marie-Françoise Raynal, Patricia Fromont, Jean-Yves Muller, Danielle Rebibo, Léna Absi, Olivier Garraud

**Affiliations:** 1 Etablissement Français du Sang Auvergne Loire France; 2 Centre Hospitalier de Montluçon Montluçon France; 3 Centre Hospitalier de Vichy Vichy France; 4 Laboratoire d'Immunologie CHU de Nantes Nantes France; 5 Etablissement Français du Sang Direction Médicale et Scientifique Paris France

## Abstract

TRALI is considered a serious hazard among immune complications of blood transfusion and its occurrence is admitted to be globally underestimated. Each type of blood product is likely to cause TRALI. We report here on two consecutive observations of TRALI caused by red blood cell concentrates, in which anti-HLA class I and class II antibodies resulting from post-gravitational allo-immunization were evidenced in donors. HLA class I and II antigenic community between recipients and donors' husbands were found and strong reacting IgG antibodies directed at several of those common antigens were detected in the donors' serum. Both donors had more than 3 pregnancies, raising the issue of blood donor selection or of plasma reduction for cellular products.


*Clinical & Developmental Immunology*, December 2005; 12(4): 243–248

Taylor & Francis

Taylor & Francis Group

### Transfusion related acute lung injury (TRALI) caused by red blood cell transfusion involving residual plasma anti-HLA antibodies: A report on two cases and general considerations

HÉLÈNE ODENT-MALAURE[1], FABIENNE QUAINON[1], PASCALE RUYER-DUMONTIER[1], SOIZICK DUCROZ[2], PHILIPPE VERDIER[2], EVELYNE VOITELLIER[3], MARIE-FRANÇOISE RAYNAL[3], PATRICIA FROMONT[4], JEAN-YVES MULLER[4], DANIELLE REBIBO[5], LÉNA ABSI[1], & OLIVIER GARRAUD[1]

[1]*Etablissement Français du Sang Auvergne, Loire, France,* [2]*Centre Hospitalier de Montluçon, Montluçon, France,* [3]*Centre Hospitalier de Vichy, Vichy, France,* [4]*Laboratoire d'Immunologie, CHU de Nantes, Nantes, France, and* [5]*Etablissement Français du Sang, Direction Médicale et Scientifique, Paris, France*

**Abstract**

TRALI is considered a serious hazard among immune complications of blood transfusion and its occurrence is admitted to be globally underestimated. Each type of blood product is likely to cause TRALI. We report here on two consecutive observations of TRALI caused by red blood cell concentrates, in which anti-HLA class I and class II antibodies resulting from post-gravitational allo-immunization were evidenced in donors. HLA class I and II antigenic community between recipients and donors' husbands were found and strong reacting IgG antibodies directed at several of those common antigens were detected in the donors' serum. Both donors had more than 3 pregnancies, raising the issue of blood donor selection or of plasma reduction for cellular products.

### Introduction

There are currently no alternatives to blood transfusion (BT). Many attempts have been made, which were primarily motivated by: (i) a desire to secure supplies in case of catastrophic circumstances or for use in the battle field and (ii) the desire to reduce or eliminate the occurrence of infectious consequences of blood transfusion, such as those encountered in the early (human immunodeficiency virus (HIV)) and late (hepatitis C virus (HCV)) 1980s. Although, some risks regarding blood transfusion still exist due to possible emerging diseases, which may be transmissible via transfusion, the overall risk from transfusion transmitted (TT) infections is currently very low. Incidental exposure to major TT viruses, such as HIV and HCV, are very rare. However, further measures can be taken for hepatitis B virus (HBV), cytomegalovirus (CMV), erythrovirus (parvovirus) B19, parasites and bacteria (the most current TT infection source) (Koistinen 2004). However, the immunological risks linked to bone marrow transfusion (BMT) remain quite high, although these are diverse in terms of causes and consequences (Goodnough 2003).

Blood or blood product substitutes have been developed, but have consistently resulted in an immunogenic response, such as the host production of neutralizing antibodies to anti-hemophiliac recombinant factor VIII (Key 2004). Among the immunological risks presented by transfusion of labile blood products, such as packed red blood cells (PRBC), fresh frozen therapeutic plasma and platelet concentrates, is transfusion related acute lung injury (TRALI), a transfusion-associated acute respiratory distress syndrome (ARDS). It is now commonly thought that TRALI is largely underestimated both in frequency and in diagnosis (according to the "iceberg theory" of the clinical forms) (Boshkov 2004). TRALI may be confused with other ARDS, such as transfusion associated circulatory overload (TACO), acute

Correspondence: O. Garraud, EFS Auvergne-Loire, 25 Avenue Pasteur, Saint-Etienne cedex 2 42023, France. Tel: 33 477 814 255. Fax: 33 477 808 294. E-mail: olivier.garraud@efs.sante.fr

ISSN 1740-2522 print/ISSN 1740-2530 online © 2005 Taylor & Francis
DOI: 10.1080/17402520500307926

pulmonary edema and cardiogenic shock. These are possible complications of BT associated with hypervolemia or overload. Other causes of ARDS that are observed following transfusion are anaphylactic allergy, severe bacterial infection and septic shock. Except for anaphylactic allergy, these syndromes are not caused by an immunological response (Popovsky et al. 1983).

The recognition of TRALI necessitated the generation of a consensus description and a commitment to understand the physiopathology of the syndrome; the committee's consensuses have clarified clinical, radiological and biological criteria describing TRALI (Goldman et al. 2005; Toy et al. 2005). TRALI presents with pulmonary endothelial lesions not associated with any other cause of ARDS or cardiogenic shock. The physiopathology of TRALI is complex and not clearly understood. TRALI presents as an accumulation of polynuclear neutrophils locally activated by antibodies in the pulmonary endothelium. The antibodies seem to activate the neutrophils, monocytes, and/or endothelial cells. Cytokines and toxic cellular products of platelet lipid origin may amplify these acute inflammatory responses. However, labile blood products other than platelets can also cause TRALI. The patient may also have a clinical predisposition for TRALI. In any case, it seems that there is an antigen (Ag)-antibody reactivity between the blood donor's plasma and the recipient's cells, or possibly vice-versa in some cases. Even a residual amount of antibody-containing plasma from the donor, which we describe here, can be responsible for TRALI (Popovsky 2000; Kopko 2004a,b; Nishimura et al. 2004; Muller 2005). The pathophysiology of TRALI appears to result from (i) the transfer of pre-existing antibodies from the blood donor to the recipient, (ii) a clinical predisposition or (iii) the involvement of metabolic factors originating from the blood product (Popovsky et al. 1983; Popovsky and Moore 1985; Popovsky and Haley 2001; Boshkov and Silliman 2004).

The antibodies are principally derived from the donor plasma and are primarily of the IgG isotype. The antibodies recognize targets on the recipient's white blood cells (WBCs), including HLA class I or II, granulocyte antigen (commonly referred to as anti-HNA antibodies), and the CD16 surface antigen. The activated WBC contribute to lung epithelium damage (Kopko and Popovsky 2004). Pregnancies are thought to be the origin of such allo-antibodies. Individuals with a history of transfusion, as well as graft or transplantation, cannot donate blood.

TRALI occurrences could be largely underestimated. It is currently estimated that one in five described TRALI incidents has a fatal outcome (Wallism 2003). We present here two consecutive occurrences of TRALI, which were observed after BT of PRBC and mediated by traces of anti-HLA antibodies present in the residual plasma.

### Patients observations and laboratory investigations

The Auvergne-Loire regional haemovigilance department of the Établissement Français du Sang over the last 4 years has identified three cases of TRALI diagnosed according to the criteria established by Popovsky and Moore (1985). Approximately 330,000 blood products were distributed during this period. The first case of TRALI was reported and involved transfused plasma products (Odent-Malaure 2001; Fromont et al. 2002; Odent-Malaure 2004). The two remaining cases are presented here and involved transfused PRBC. The two cases will be referred to as case 1 and 2.

### *Case 1*

A 78 year-old male presented with myelodysplasia in June 2003 and received one PRBC (ABO, RH1, 2–5 and RH-KEL1 and two compatible). There were no irregular antibodies detected by indirect antiglobulin test (IAT) immediately prior to transfusion. The transfusion-related accident occurred immediately, after three quarters of the transfusion, and was diagnosed as TRALI based on clinical and radiological symptoms; the patient developed an acute dyspnea with bilateral crackles during lung auscultation, chest X-ray examination suggested a diffuse interstitial pulmonary edema, and the diagnosis of acute respiratory distress was confirmed by laboratory tests. Resuscitation measures were taken and the patient recovered from this episode within 24 h. The PRBC was 7 day old and had been prepared from blood donated by a 36 year-old female that had given birth to 4 children (5 total pregnancies). Donors should not have been previously transfused according to national French regulations. A standard micro-lymphocytotoxicity technique (MLCT) determined that the donor's serum contained strongly reacting anti-HLA class I and anti-HLA class II IgG antibodies (93% of the panel cells were labeled). (Lazezari et al. 1976). It was difficult to identify the antigens using either classical MLCT or a sensitive LAT® (Lambda Antigen Tray- class I, class II, One-Lambda, Canoga Park, CA, USA) technique. We looked for shared HLA specificities between the recipient's and the donor's (patient's husband) peripheral blood mononuclear cells. We identified four shared Ag, HLA A9, B12, DR7 and DR53, using micro-lymphocytotoxicity, serology and molecular biology techniques, which have been validated by the European Foundation for Immunogenetics (Daniels 2004; Turner 2004).

PCR sequence specific primer genotyping was performed using the Dynal (Biotech ASA, Oslo, Norway) kits for HLA class I Ag and One Lamba kits for the HLA class II Ag. Anti-HLA B12 (B44 + B45) and DR53 were identified in the donor's serum by the LAT® technique. The serum IgG antibody reacted strongly and remained granulo-agglutinating at a titer of 1–64. Furthermore, the presence of additional anti-granulocyte antibodies was suspected because the donor's serum proved capable of reacting, both in flow cytometry and in the Lazezari's microagglutination technique (GAT), with HNA1a, HNA1b, HNA1c, and HNA2a granulocytes (Lazezari et al. 1976; Veys et al. 1989; Bux et al. 1993). Monoclonal antibody (mAb)-specific immobilization of granulocyte antigens (MAIGA) test positivity indicated the presence of specific anti-granulocyte antibody because the donor serum gave a weak positive reaction with mAb DJ-130c (Dakocytomation, Trappes, France), but not mAb 3G8 (Beckton Dickinson, Le Pont-de-Claix, France). MAb DJ-130c occasionally gave unexplained positive reactions with HLA class I antibodies in the absence of any anti-granulocyte antibody (JYM's unpublished observations). Thus, the involvement of an anti-HNA antibody was not considered. The observed granulocyte reactivity was determined as an anti-HLA class I antibody. Case 1 is summarized in Table I.

*Case 2*

A 70 year-old female underwent surgery for colonic carcinoma and received two consecutive PRBC to compensate for anemia in March 2004. This unmarried patient did not report any previous pregnancy and had not been previously transfused or allografted. No irregular antibodies were detected by pre-transfusion IAT. Two ABO, RH1, 2–5, and RH-KEL1 and 2 compatible leukocyte-reduced PRBC were transfused. The first one was prepared from a 30 year-old female donor reporting 3 children (2 pregnancies of which one was gemellar). The second PRBC was prepared from a 42 year-old female reporting 4 children (5 pregnancies in total). These PRBC were stored for 32 and 39 days prior to infusion, respectively. The reaction occurred 2 h after the end of the second PRBC infusion. It was determined as TRALI based on clinical and radiological signs: the patient reported chills and acute dyspnea developed. Examination showed diffuse bilateral crackles without wheezing. A radiograph of the chest revealed diffuse, bilateral air-space consolidation, which was consistent with the presence of pulmonary edema. The diagnosis of acute respiratory distress was confirmed by laboratory tests. The patient was transferred to an intensive care unit and recovered from this episode within 48 h.

The recipient's serum had neither anti-HLA class I nor II antibodies prior to transfusion, which was determined by retesting of the pre-transfusion blood sample that was kept frozen. Antibodies were found only in the second donor's serum. This serum reacted with the recipient's and her current husband's T- and B-lymphocytes, which were prepared after indirect magnetic separation of ficoll isolated PBMC

Table I. Case report 1 on the occurrence of TRALI after transfusion of packed red blood cells.

| | Blood donor | Immunizer | Blood receiver |
|---|---|---|---|
| Age | 36 yo | | 78 yo |
| Gender | Female | M (Spouse) | Male |
| Medical history | | | |
| Indication of transfusion | | | Myelodysplasia (anemia) |
| Occurrence of the adverse reaction | | | During (3/4) the transfusion* |
| Outcome | | | Favorable within 24 h |
| Immunization | | | |
| Transfusion | None | | Previously transfused (4 times in two years) |
| Pregnancies | | | |
| No. of pregnancies | 5 (4 children) | | NA |
| Serology | | | |
| Anti-HLA Abs† | Strongly reacting anti-class I and II Abs (anti-A29, B44 + 45 (12) DR7 | NT | Not tested prior to the transfusion |
| | | | None immediately after transfusion |
| Anti-HNA Abs‡ | Suspected, not confirmed | NT | None |
| HLA-typing (a) | | | |
| HLA A | 25 (10), 30 (19) | 24 (9), 29 (19) | 23 (9), 24 (9) |
| HLA B | 18, – | 44 (12), 45 (12) | 8, 44 (12) |
| HLA DR | 2, 3 | 7, 10 | 0103, 7 |
| HLA DQ | 1, 2 | 1, 2 | 1, 2 |
| Shared Ags | – | (A9), B12, DR7, | |
| Ab production to | (A29), B12, DR7 | – | – |

*The first PRBC was transfused. This pack was issued from a donation from a 36 year-old (yo) female reporting 3 pregnancies. This female donor tested negative for anti-HLA and HNA plasma antibodies.

†Testing by standard Micro-Lymphocytotoxicity (MLCT) and by ELISA (LAT®; Lambda Antigen Tray-class I and II, One Lambda, Canoga Park, CA, USA). For MLCT, T- and B-cells were prepared by indirect magnetic separation of ficoll-isolated PBMCs23 (Dynabeads, Dynal, Roskilde, Denmark).

‡Testing by the MAIGA technique, using a panel of anti-CD16 and -CD177 monoclonal antibodies (Abs) (Bux et al.).

(Dynabeads®, Dynal, Roskilde, Denmark). Anti-HLA-A29, -B17, -B12 (B44 + B45) and -DR7 allo-antibodies were identified in this donor's serum. The donor's husband, typed for HLA class I and II, was A2, A29, B44, B51, DR7, DQ2 and DQ3. The recipient typed positive for DR7 class II Ags and for B45 class I Ag, which along with B44 are the two main serologic splits of B12. B12 Ags are known to strongly cross-react in terms of antigenicity and immunogenicity. This anti-class I specificity could account for the granulocyte reactivity observed with this serum using GAT and flow cytometry. MAIGA negativity using anti-CD16 and anti-CD177 mAbs suggested there was no additional specific anti-granulocyte reactivity. Case 2 is summarized in Table II.

### Discussion

These two consecutive cases underscore the role of PRBC in TRALI in addition to the more commonly reported occurrences involving platelets and plasma. TRALI has been suspected or confirmed in over 10% of transfusion-related deaths in each of the last 4 years, and in over 15% of these cases over the last 2 years in the U.S. (America's Blood Center 2004). According to a consensus at a Canadian conference held earlier in 2004, the estimate for TRALI occurrence ranges from 1 in 5000 to 1 in 100,000 for all transfusion components. However, this depends on what is actually defined as TRALI (Mariani 2003). In general, the occurrence of TRALI has been largely under-estimated and the relevant physiopathology is still not understood. Furthermore, data establishing a definitive link between gender and anti-HLA antibodies, a major cause of TRALI is lacking. Fifteen cases of TRALI were recorded and fully documented in 2003 according to the French Haemovigilance Network at the Etablissement Français du Sang. Three were associated with fatalities. Nine, which included all 3 fatal cases, were associated with platelet concentrates (incidence: 1/25,000 products), 2 were associated with therapeutic plasma (incidence: 1/130,000) and 4 were associated with PRBC (incidence: 1/500,000) (Rebibo et al. 2004). These frequencies are comparable to those from the most organized and centralized haemovigilance systems, such as the British (SHOT) and the Canadian systems. The estimated mortality rate ranges from 10 to 20%, provided that the case is identified. Thus, it is likely that TRALI is more important than initially thought.

We report here two occurrences of clinically documented TRALI that resulted from PRBC transfusion,

Table II. Case report 2 on the occurrence of TRALI after transfusion of packed red blood cells.

| | Blood donor | Immunizer | Blood receiver |
|---|---|---|---|
| Age | 30 yo | | 72 yo |
| Gender | Female | M (Spouse) | Female |
| Medical history | | | |
| Indication of transfusion | | | Colonic carcinoma necessitating surgery (anemia) |
| Occurrence of the adverse reaction | | | Two h after the end of the second PRBC* |
| Outcome | | | Favorable within 48 h |
| Immunization | | | |
| Transfusion | None | | 1st occurrence |
| Pregnancies | | | |
| # of pregnancies | 3 (1 gemellar) | | None |
| Serology | | | |
| Anti-HLA Abs† | Anti-A29, B17, B44 + B45 (12), DR7† | NT | None prior to the transfusion<br>None after transfusion (confirmed) |
| Anti-HNA Abs‡ | None | NT | NT |
| HLA-typing (a) | | | |
| HLA A | 3, – | 2, 29 (19) | 3, 26 (10) |
| HLA B | 7, – | 44 (12), 51 (5) | 8, 45 (12) |
| HLA DR | 2, 13 (6) | 7, – | 3, 7 |
| HLA DQ | 1, – | 2, 3 | 2, – |
| Shared Ags | – | B44/45 (12), DR7 | |
| Ab production | (A 29), B44/45 (12), DR7 | – | – |

*The first PRBC was transfused. This pack was issued from a donation from a 30 year-old (yo) female reporting 3 pregnancies. This female donor tested negative for anti-HLA and HNA plasma antibodies.

†Testing by standard Micro-Lymphocytotoxicity (MLCT) and by ELISA (LAT®;Lambda Antigen Tray-class I and II, One Lambda, Canoga Park, CA, USA). For MLCT, T and B cells were prepared by indirect magnetic separation of ficoll-isolated PBMCs (Dynabeads, Dynal, Roskilde, Denmark).

‡Testing by the MAIGA technique, using a panel of anti-CD16 and -CD177 monoclonal antibodies (Abs) (Bux et al.).

but ended with favorable outcomes. The clinical details have been presented elsewhere (Odent-Malaure 2004). Tables I and II provide the biological details and procedures. It is worth noting that in the two cases reported here the PRBC bags came from two different manufacturers. The residual volumes of plasma, shown to contain anti-HLA antibodies, were 32 and 39 ml and contained approximately 50 g free soluble protein per liter of plasma. Leuko-reduction was performed during the processing to leave less than $10^6$ leukocytes per unit in accordance to the national French requirements. Residual leukocytes were $\leq 9$ and $\leq 7 \times 10^4$ per unit after preparation, but it is unlikely that the leukocytes persisted after a 37 day storage at 4°C (Case 2). One PRBC sample was 7 day old (Case 1), while the other was 37 day old (Case 2), which likely excludes the role of blood product aging in PRBC associated TRALI (Popovsky 2000). The two adverse reactions occurred using blood products donated by multiparous females. The cases involved the development of strong allo-antibodies (IgG) against HLA class I and II Ags, which were present on the somatic cells of the donors' husbands and were shared by the respective recipients. Interestingly, HLA typing of the donors' husbands proved useful in identifying the specificity involved in TRALI. Such allo-immunization is very likely to be related to pregnancies and the donor's husband's HLA typing may be useful for identifying the specificity involved in TRALI as illustrated by Case 1 (Popovsky and Davenport 2001). It is noteworthy that in each case the outcome for the patient was favorable. Based on these observations, and those in the general literature, no conclusions regarding the selection of blood donors can be made. Systematic exclusion of multiparous donors would lead to a dramatic shortage of cellular blood products (Muller et al. 1981). Large scale testing of multiparous female donor serum for anti-HLA antibodies after each pregnancy may be possible, but after which pregnancy to begin such testing remains to be determined. Indeed, it is estimated that among the 332,240 whole blood donations 16,500 PRBC were prepared and distributed from donations by females with greater than 3 children or pregnancies during the survey period in the Auvergne-Loire region at Etablissement Français du Sang. Implementation of cost-effective techniques leading to an almost complete depletion of residual plasma in PRBC might be a desirable alternative to exclusion without or after testing of female donors having had a critical number of pregnancies.

Despite great progress with regard to the reduction in pathogen-induced complications of transfusion, a risk of immunological complications resulting from the infusion of trace amounts of adverse antibodies remains. The selection of donors merits special focus, especially for blood recipients with sensitive medical, such as multiple transfusions, or surgical histories.

### References

America's Blood Center 2004. PBAC recommends more research before TRALI interventions. ABC Newsletter July 30 :6.

Boshkov LK, Silliman CC. 2004. Transfusion related acute lung injury. Blood Therapies Med 4:85–93.

Bux J, Kober B, Kiefel V, Mueller-Eckhardt C. 1993. Analysis of granulocyte-reactive antibodies using an immuno-assay based upon monoclonal antibody-specific immobilization of granulocyte antigens. Transf Med 3:157–162.

Daniels G. 2004. Molecular blood grouping. Vox Sang 87:63–66.

Fromont P, Goyenaga MH, Odent-malaure H, Lamy B, Bignon JD, Valentin N, Muller J-Y. 2002. Role of anti-HLA-B51 agglutinating antibody in the occurrence of a transfusion related acute lung injury (TRALI). In: Borzini P, editor. (Oral communication) Proceedings of the 7th European Symposium on Platelet, Granulocyte and Red Cell Immunology. Milano: Simiti Servizi.

Goldman M, Webert KE, Arnold DM, Freedman J, Hannon J, Blachjman MA. 2005. TRALI Consensus Panel. Proceedings of a consensus conference: Towards an understanding of TRALI. Transfus Med Rev 19:2–31.

Goodnough LT. 2003. Risks of blood transfusion. Crit Care Med 31:S678–S686.

Key NS. 2004. Inhibitors in congenital coagulation disorders. Br J Haematol 127:379–391.

Koistinen JL. 2004. Safety aspects of blood transfusion and European legislation. Hematol J S3:S83–S86.

Kopko PM, Popovsky MA. 2004. Pulmonary injury from transfusion-related acute lung injury. Clin Chest Med 25:105–111.

Kopko PM. 2004. Leukocyte antibodies and biologically active mediators in the pathogenesis of transfusion-related acute lung injury. Curr Hematol Rep 3:456–461.

Kopko PM. 2004. Review: Transfusion-related acute lung injury: Pathophysiology, laboratory investigation and donor management. Immunohematol 20:103–111.

Lazezari P, Jiang A, Lees S. 1976. A microagglutination technique for the detection of granulocyte alloantibodies. NIAID manual: A tissue typing technique, NIAID (Bethesda, Md), p 4–6.

Mariani SM. 2003. Conference report—Transfusion and TRALI: What are the risks today? Highlights from the 71st annual meeting of the American Society for Clinical Laboratory Science. July 22-26, Philadelphia, Pennsylvania. Med Gen Med 10:6.

Muller JY, Cartron JP, Trillot P, Halle L, Roquin H. 1981. Platelet transfusions: Automated selection of donors. Transfusion 21:261–267.

Muller JY. 2005. TRALI. Transfus Clin Biol 12:230–235.

Nishimura M, Ishikawa Y, Satake M. 2004. Activation of polymorphonuclear neutrophils by immune complexes: Possible involvement in development of transfusion related acute lung injury. Tranfus Med 14:359–367.

Odent-Malaure H, Gallon Ph, Tridon A, Fouilhoux AC, Benamara A, Lamy B, Le Petit J.-C. 2001. Transfusion-related lung injury (TRALI) during plasmapheresis and transfusion (oral communication). Proceedings of VIIth European Congress of the International Society of Blood Transfusion, Paris, 15–18 July.

Odent-Malaure H. 2004. Cas de TRALI rapportés par les regions tranfusionnelles d'Auvergne-Loire, Nord de France, Bourgogne-Franche-Comté et Ile-de France. Bull Hémovigilance/AFSSaPs 8:4.

Popovsky MA, Davenport RD. 2001. Transfusion-related acute lung injury: Femme fatale? Transfusion 41:312–315.

Popovsky MA, Haley NR. 2001. Further characterization of transfusion-related acute lung injury: Demographics, clinical and laboratory features and morbidity. Immunohematology 17:157–159.

Popovsky MA, Moore SB. 1985. Diagnostic and pathogenetic consideration in transfusion-related acute lung injury. Transfusion 25:573–577.

Popovsky MA. 2000. Transfusion-related acute lung injury. Curr Opin Hematol 7:402–407.

Popovsky MA, Abel MD, Moore SJ. 1983. Transfusion-related lung injury associated with passive transfer of antileukocyte antibodies. Am Rev Resp Dis 128:185–189.

Rebibo D, Hauser L, Slimani A, Herve P, Andreu G. 2004. The French haemovigilance system: Organization and results for 2003. Transfus Apheresis Sci 31:145–153.

Toy P, Popovsky MA, Abraham E, Ambruso DR, Holness IG, Kopko PM, McFarland JG, Nathens AB, Silliman CC, Stroncek D. 2005. National heart, lung and blood institute working group on TRALI. Transfusion-related acute lung injury: Definition and review. Crit Care Med 33:721–726.

Turner D. 2004. The human leukocyte antigen (HLA) system Vox Sang 87 S1:87–90.

Veys PA, Gutteridge CN, Macey M, Ord J, Newland AC. 1989. Detection of granulocyte antibodies using flow cytometric analysis of leukocyte immunofluorescence. Vox Sang 56:42–47.

Wallis JP. 2003. Transfusion related acute lung injury—under-diagnosed and under-reported. Br J Anaesth 90:573–576.

